# Nutritional components and protein quality analysis of genetically modified phytase maize

**DOI:** 10.1080/21645698.2021.2009418

**Published:** 2022-02-01

**Authors:** Yichun Hu, Liqin Linghu, Min Li, Deqian Mao, Yu Zhang, Xiaoguang Yang, Lichen Yang

**Affiliations:** a Key Laboratory of Trace Element Nutrition of National Health Commission, National Institute for Nutrition and Health, China CDC, Beijing, China; b Prevention and Control of Chronic Diseases and Student Health Care, Shanxi Center for Disease Control and Prevention, Taiyuan, Shanxi, China

**Keywords:** Phytase maize, nutritional components, Bama pigs, protein digestibility, amino acid score

## Abstract

The nutritional components and protein quality of genetically modified maize expressing phytase gene (GM) were analyzed and evaluated in this study. The nutritional components were analyzed by Chinese national standard methods. The ileostomy Bama miniature pigs were utilized to analyze the true digestibility of protein and amino acids. The digestible indispensable amino acid score (DIAAS) was adopted to evaluate the protein quality of GM, its parental maize (PM) and commercial available maize Zhengdan 958 (ZD). Meanwhile, the widely used protein digestibility corrected amino acid score (PDCAAS) was also calculated and compared with DIAAS. The content of protein, fat, vitamins, and minerals of all the strains of maize are in the normal ranges of OECD and/or ILSI. The DIAAS of GM, PM, and ZD were 54.57, 31.75, and 33.91, respectively, and the first limiting amino acid for GM, PM, and ZD was lysine. In conclusion, the introduction of *phyA2* gene in GM maize does not disturb the digestion of protein/amino acid, but has the ability to promote the digestion of amino acids.

## 1. Introduction

Maize (*Zea mays*) is the most widely distributed crop in the world, and also one of the most important food and feed crop in China. It is reported that about 80% production of maize was currently used in feed industry in China.^1^ Phosphorus (P) is an essential element for animal growth. Phosphorus in plant seeds is mainly stored in the form of phytic acid. Phytic acid has a great adhesion affinity to cations such as calcium, magnesium, iron and zinc, and forms insoluble phytate in the solution.^2,3^ This leads to micronutrient malnutrition in people whose staple food is grains and legumes.^4^ Maize is rich in phosphorus, about 60%–80% of it exists in the form of phytate.^5^ Pig, poultry and other monogastric animals are difficult to utilize phytic acid due to the lack of enzyme to decompose it, and the general utilization rate is only 0%–40%.^6^ In addition, undigested phytate in animal manure is considered as a major source of phosphorus pollution to the environment from agricultural production. Under this circumstance, phosphate supplementation is required for optimal animal growth. Phytase can not only effectively reduce the chelating of phytic acid to protein,^7^ but also improve the utilization of protein, phosphorus and other mineral elements. In order to solve the problem of phosphorus utilization, the traditional method is to add phytase produced by microbial fermentation in the process of feed preparation, but it is expensive and risk of instability. Utilizing ideal phytase to obtain genetically modified plants that can express enough phytase by genetic engineering technology seems to be a more economical and effective way to solve the above problems. In 2001, Richardson et al. transferred the phytase gene from *Aspergillus Niger* into Arabidopsis thaliana, where it was constitutively expressed in the root system and secreted outside the cell.^8^ The phytase activity in the root tissue and root exudates of the transgenic plant was significantly increased.^9^ The phytase gene maize (GM) analyzed in this study was transformed from the *phyA2* gene obtained from *Aspergillus niger* into maize through particle bombardment.^10^ The phytase gene *phA2* is a gene with independent intellectual property rights in China.^9^ Tobacco and Arabidopsis were the mainly model plants to improve the ability of plants to absorb and utilize phosphorus with the help of genetically modified technology. Phytase gene was rarely transferred into crops before the materials analyzed in this study were developed, especially in maize. The phytase protein was specifically expressed in the seeds rather than other tissues to a concentration of 0.5 g/100 g seeds. The phytase maize was developed to improve the utilization rate of phosphorus, calcium and other elements, and it effectively reduces the cost of feed production. The phytase maize has obtained the safety certificate of agricultural genetically modified organisms (production application in Shandong province)^11,12^ by strict safety assessment. The application prospects and effects of the phytase maize have been widely affirmed.^13^ Nevertheless, its safety and nutritional aspects are widely concerned due to the use of genetically modified technology. Several reports on the phytase maize mainly focused on its toxicological evaluation,^14^ but there are few reports on its nutritional evaluation.

Zhengdan 958 maize (ZD) is the largest variety in China in terms of cultivated acreage and yield. It is the most excellent high-yield variety in China because of its good resistance, high seed setting rate, drought resistance and high-temperature resistance. In recent years, the annual planting area has reached more than 4 million hectares, accounting for about 10% of the total planting area.^15,16^ Therefore, it is usually used as the parent variety of genetically modified maize or a routine control to compare the quality, resistance, yield, adaptability, etc., in China.

Since the expression product of the transferred gene in the plant is protein, the focus of nutrition and safety evaluation is on the change of protein. In terms of nutritional evaluation, in addition to nutritional components, researchers are more focusing on the digestion and metabolism of proteins and amino acids of genetically modified products in vivo. The purpose of this study is to make nutritional evaluation for the genetically modified phytase maize by analyzing the nutritional composition and evaluating the protein quality by amino acid score through digestibility experiment in Bama miniature pigs. On one hand, we analyzed the effect of introduced exogenous gene *phyA2* on the nutrients’ components (protein, fat, carbohydrate, vitamins, minerals, etc.) in maize by comparing the phytase maize with its parental maize and commercialized maize ZD. On the other hand, among these nutrients, we are most concerned about the change of protein (amino acids) content and their digestion and metabolism in pigs, because of the introduction of exogenous gene.

## 2. Materials and Methods

### 2.1 Materials

By use of Gene-gun method, the *phyA2* gene derived from *Aspergullus Niger* was transformed into its traditional parental maize (PM) by Biotechnology Research Institute, Chinese Academy of Agricultural Sciences. The PM, GM and ZD were all provided by Beijing Origin Agritech Limited. All the maize samples were harvested, dried and ground to a 0.5-mm mesh screen for the year of the experiment. The nutritional components’ analysis and feed of digestibility experiment were then carried out.

### 2.2 Nutrients Components Analysis

The nutritional components of the maize samples were determined by Beijing Institute of Nutrition Sources (National Laboratory Certification CNAS No. L2678, Measurement Certificate No. 2004010329Z). The protein,^17^ fats,^18^ carbohydrates,^19^ fiber,^20^ water,^21^ ashes,^22^ amino acids,^23^ vitamin A and vitamin E,^24^ vitmian B1,^25^ vitamin B2,^26^ vitamin B6,^27^ vitamin B12,^28^ niacin,^29^ chromium,^30^ phosphorus,^31^ potassium, magnesium, calcium, iron, zinc, sodium^32^ were determined by Chinese national standard method. Phytate phosphorus was detected by the method described by Chen RM.^5^

### 2.3 Digestibility Experiment

10 healthy castrated male Bama miniture pigs (Beijing Tong He Sheng Tai Institute of Comparative Medicine, license: SCXK (Beijing), 2015–0004), were individually housed in stainless-steel cages in the Institute of experimental animals, Chinese Academy of Sciences (temperature 20°C to 25°C, humidity 40% to 70%). All Bama pigs (BMPs) were surgically fitted with post-valve T-intestinal cannulas after 36 h fasting and 12 hours’ water banning after 7 days adaptive feeding.^33^ Eight BMPs, which were well recovered from the surgery and with good appetite after 2 weeks’ full recovery, were selected. Under the premise of meeting the nutritional needs of BMPs, all the feed in this experiment were prepared on the principle of maximum incorporation of maize. Non-transgenic soybean oil was added to the feed to increase the taste, vitamins and minerals were added to meet the growth needs of BMPs, and chromium trioxide was added as the indicator. The content of added maize in feed were 93.84%, 93.81% and 93.73% for GM feed, PM feed and ZD feed respectively (The formula of feed, Appendix 1). The 5% casein feed was served to determine the endogenous amino acid losses (EAL). Normal commercial feed (Beijing Tonghe Ecological Co., Ltd) was used for recovery of different feed intervals. A replicated latin square design was applied for GM, PM, ZD, and casein comparison group (The experimental arrangement, Appendix 2). During the feeding period of each feed, the ileal chyme was continuously collected and frozen at −20°C for 3 days (from 8:00 to 20:00). The ileal chyme of each pig in each collection period was mixed respectively, freeze-dried and crushed through 60 mesh for nutrients’ detection. Blood was taken from anterior vena cava before and after digestibility experiment to determine blood biochemistry and blood routines. Blood routines were measured by automatic hematology analyzer (XT-1800IV, SYSMEX), and blood chemistry was measured by an automatic biochemical analyzer (7080, HITACHI). All operations during the experiment meet the requirements of animal welfare, and the experimental scheme was approved by the ethics committee of the National Institute for Nutrition and Health, China Center for Disease Control and Prevention.

### 2.4 Protein Digestibility and Amino Acid Score

The digestible indispensable amino acid score (DIAAS) was adopted to evaluate the protein quality of phytase maize, and the commonly used protein digestibility corrected amino acid score (PDCAAS) were also used and compared with DIASS in this study.

The true digestibility (TD) of GM, PM, and ZD were obtained from the digestibility experiment and calculated according to equation (1), (2) and (3).^34^ The DIAAS of GM, PM, and ZD were calculated according to equation (1), (2), (3) and (5). ^35^ The PDCAAS of GM, PM, and ZD were calculated according to equation (1), (2), (3), (5) and (6). ^36^ The scoring model of reference protein was the scoring model for adult (aged above 18) revised by the FAO/WHO/UNU expert panel in 2007.^37^

(1) $$AD\left(\%\right)=100-\frac{Cr_{diet}}{Cr_{chyme}}\times \frac{N_{chyme}}{N_{diet}}\times 100$$

(2) $$EAL\left(g/kg\right)=N_{chyme}\times \frac{Cr_{diet}}{Cr_{chyme}}$$

(3) $$TD\left(\%\right)=AD+\frac{EAL}{N_{diet}}\times 100$$

(4) $$DIAAS\left(\%\right)=\frac{\begin{array}{c} Content\;of\;digestible\;essential\;\\ amino\;acid\;in\;measured\;protein \end{array}}{\begin{array}{c} Content\;of\;digestible\;essential\;\\ amino\;acid\;in\;refernce\;protein \end{array}}\times 100$$

(5) $$AAS\left(\%\right)=\frac{\begin{array}{c} Content\;of\;essential\;amino\;\\ acid\;in\;measured\;protein \end{array}}{\begin{array}{c} Content\;of\;essential\;amino\;\\ acid\;in\;reference\;protein \end{array}}\times 100$$

(6) $$PDCAAS\left(\%\right)=AAS\times True\;Digestibility\;of\;protein$$

where AD is apparent digestibility, TD is ture digestibility, EAL is endogenous amino acid loss, Cr_diet_ is the content of chromium in diet (mg/kg), Cr_chyme_ is the content of chromium in chyme (mg/kg), N_diet_ is the content of nutrients in diet (g/kg), and N_chyme_ is the content of nutrients in chyme (g/kg).

### 2.5 Statistical Analysis

All the data collected were analyzed using SAS 9.4 software (SAS Institute Inc., Cary, NC, USA). All the data were presented as group mean values ± standard deviation (mean ± SD), continuous data were compared with ANOVA, and frequency data were compared using a chi-square test. The difference was statistically significant with *P*<.05.

## 3. Results

### 3.1 Nutritional Components of Phytase Maize

The protein, fat and fiber were within the normal reference range of Organisation for Economic Cooperation and Development (OECD)^38^ and International Life Sciences Institute (ILSI).^39^ The carbohydrate of PM and ZD was lower than the OECD range and ILSI range. The proportion of the 18 amino acids listed in Table 1 to protein was 91.80% in GM, 84.70% in PM and 93.14% in ZD, respectively. The threonine, glycine, and proline were slightly lower than the OECD reference normal range, but they were all within the range of ILSI reference normal range. The isoleucine of ZD was slightly lower than the reference ranges (0.18 ~ 0.69 g/100 g). All the other amino acids were within the range of OECD and ILSI.

**Table 1.** Content of main nutrients and amino acids in GM, PM and ZD maize^*.^

Component	Content			OECD range^a^	ILSI range^b^
	GM	PM	ZD		
Protein(g/100 g)	8.92	9.22	8.30	6.0–12.7	5.72–17.26(10.20)
Fat (g/100 g)	4.95	3.89	5.01	3.1–5.8	1.37–7.83(3.79)
Carbohydrate (g/100 g)^c^	78.99	72.92	73.26	82.2–82.9	77.4–89.7(84.6)
Fiber (g/100 g)	12.91	13.47	12.25	8.3–11.9	5.8–35.3(13.66)
**Amino acids** (g/100 g)					
Essential amino acids					
Threonine	0.27	0.26	0.26	0.27–0.58	0.17–0.67(0.36)
Valine	0.37	0.35	0.36	0.21–0.85	0.27–0.86(0.47)
Methionine	0.13	0.14	0.15	0.10–0.46	0.11–0.47(0.21)
Isoleucine	0.19	0.18	0.17	0.22–0.71	0.18–0.69(0.36)
Leucine	1.12	1.11	1.06	0.79–2.41	0.60–2.49(1.28)
Phenylalanine	0.42	0.41	0.39	0.29–0.64	0.24–0.93(0.52)
Lysine	0.27	0.25	0.25	0.05–0.55	0.13–0.67(0.29)
Tryptophan	0.07	0.06	0.07	0.04–0.13	0.03–0.22(0.07)
Nonessential amino acids					
Histidine	0.21	0.20	0.23	0.15–0.38	0.14–0.46(0.28)
Aspartate	0.62	0.58	0.55	0.48–0.85	0.30–1.21(0.66)
Serine	0.43	0.41	0.38	0.35–0.91	0.15–0.77(0.49)
Glutamate	1.75	1.68	1.64	1.25–2.58	0.83–3.54(1.92)
Glycine	0.26	0.24	0.25	0.26–0.49	0.18–0.69(0.38)
Alanine	0.75	0.72	0.68	0.56–1.04	0.40–1.48(0.77)
Tyrosine	0.21	0.20	0.19	0.12–0.79	0.10–0.73(0.35)
Arginine	0.37	0.34	0.32	0.22–0.64	0.12–0.71(0.47)
Proline	0.62	0.60	0.67	0.63–1.36	0.46–1.75(0.91)
Cysteine	0.11	0.10	0.11	0.08–0.32	0.12–0.51(0.21)

* GM,phytase gene maize; PM, traditional parental maize of GM; ZD, commercialized maize “Zhengdan 958”; All data in the table are calculated by dry weight.

^a^OECD (2002). ^b^ ILSI (2019) version 7.0, all the range was showed from min to max value, with mean in parentheses. ^c^ Carbohydrate content was obtained by calculation method.

### 3.2 Minerals and Vitamins

The potassium, vitamin E and vitamin B6 of GM, PM, and ZD were with the range of ILSI,^39^ but not in the range of OECD.^38^ The vitamin B2 was lower than the ILSI range (0.05 ~ 0.74 mg/100 g) but within the OECD range (0.025 ~ 0.56 mg/100 g) (Table 2). All the other minerals and vitamins were all within the OECD and ILSI range. The phytate phosphorus content and the proportion of phytate to total phosphorus of GM and PM were lower than that of ZD, and the GM was the lowest.

**Table 2.** Content of minerals and vitamins in GM, PM and ZD maize*

Composition	GM	PM	ZD	OECD range^a^	ILSI range^b^
Minerals					
Phosphorus (g/100 g)	0.29	0.29	0.25	0.23–0.75	0.13–0.55(0.31)
Potassium (mg/ 100 g)	213.96	233.92	223.10	320–720	181.00–603.00(365.45)
Magnesium (mg/100 g)	130.03	120.68	110.09	82–1000	59.4–194.0(119.97)
Calcium (mg/100 g)	5.67	6.55	7.32	3–100	1.18–101.00(4.21)
Iron (mg/100 g)	2.86	1.68	2.33	0.1–10	0.95–19.10(2.01)
Zinc (mg/100 g)	2.08	2.48	2.68	1.2–3.0	0.65–4.26(2.23)
Sodium	0.93	0.72	0.73	0–150	0.017–73.15(2.15)
phytate phosphorus (g/100 g)	0.02	0.03	0.13	/	/
phytate phosphorus /Phosphorus	6.92%	10.38%	52.73%	/	/
Vitamins					
Vitamin A (μg/100 g)	8.63	6.64	6.53	/	/
Vitamin E (mg/100 g)	4.96	3.97	4.93	0.30–1.21	0.18–8.99(2.72)
Vitamin B1 (mg/100 g)	0.19	0.20	0.19	0.23–0.86	0.13–4.0(0.37)
Vitamin B2 (mg/100 g)	0.03	0.03	0.03	0.025–0.56	0.05–0.74(0.20)
Vitamin B6(mg/100 g)	0.40	0.42	0.41	0.46–0.96	0.12–1.21(0.59)
Vitamin B12(μg/100 g)	0.34	0.43	0.34	/	/
Niacin (mg/100 g)	3.01	2.78	3.05	0.93–7.00	0.74–4.69(2.07)

* GM,phytase gene maize; PM, traditional parental maize of GM; ZD, commercialized maize “Zhengdan 958”; All data in the table are calculated by dry weight.

^a^OECD (2002). ^b^ ILSI (2019) version 7.0, all the range was showed from min to max value, with mean in parentheses.

### 3.3 Digestibility of Main Nutrients and Amino Acids

**Figure 1.**
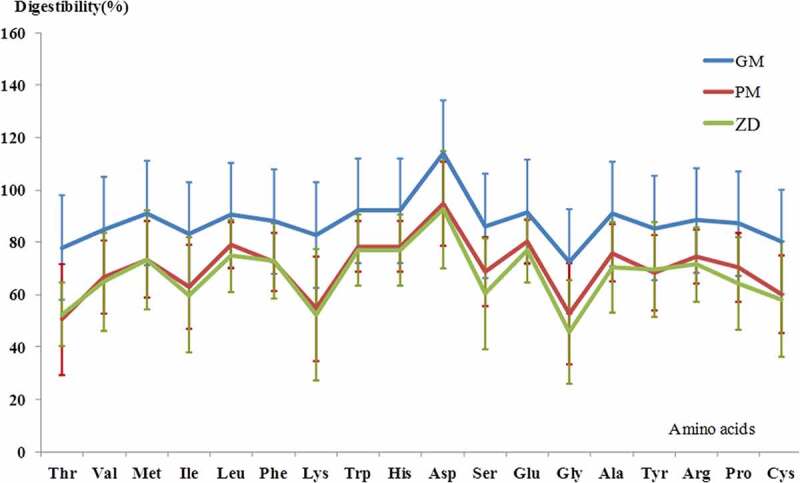
The mean ture digestibility of amino acids of GM (phytase gene maize), PM (traditional parental maize of GM) and ZD (commercialized maize “Zhengdan 958”). The first eight are essential amino acids.

All BMPs were normal in diet and water intake during the whole experiment. The digestibility of main nutritional components and amino acids are shown in Table 3. The digestibility of protein (TD), fat (AD) and carbohydrate (AD) of GM was highest, and then followed by PM and ZD, but there were no differences found in groups. The mean true digestibility of all the amino acids in GM was higher than those in PM and ZD (Table 3, Fig. 1), but not all the amino acids have significant differences in digestibility between groups. The true digestibility of lysine, histidine, serine, alanine, arginine, and proline in GM were significantly higher than that of PM and ZD (Table 3). The true digestibility of glutamate and glycine in GM was significantly higher than that of ZD. Among essential amino acids, the lowest mean digestibility was threonine in GM and PM, and lysine in ZD.

**Table 3.** Digestibility of main nutrients in GM, PM and ZD maize *

Components		Digestibility^#^ (mean ± SD, %)			P value	
		GM	PM	ZD		
Protein		101.28 ± 13.37	88.18 ± 16.35	81.84 ± 21.25	0.265	
Fat		72.94 ± 19.27	58.81 ± 23.68	55.63 ± 27.14	0.205	
Carbohydrate		88.20 ± 7.54	82.36 ± 11.54	72.27 ± 16.03	0.072	
Amino acid						
Essential amino acids						
Threonine		77.96 ± 17.59	50.52 ± 21.20	52.42 ± 12.09		0.063
Valine		84.92 ± 10.69	66.73 ± 13.83	64.96 ± 18.70		0.069
Methionine		91.13 ± 11.37	73.49 ± 14.48	73.36 ± 19.00		0.110
Isoleucine		83.08 ± 11.60	63.03 ± 16.06	59.86 ± 21.98		0.073
Leucine		90.48 ± 6.70	78.88 ± 8.82	74.83 ± 13.83		0.052
Phenylalanine		87.95 ± 7.86	72.50 ± 11.28	72.96 ± 14.49		0.064
Lysine		82.80 ± 11.22^ab^	54.72 ± 19.96	52.33 ± 25.03		0.033
Tryptophan		114.07 ± 17.12	92.28 ± 17.56	86.63 ± 28.67		2.559
Nonessential amino acids						
Histidine		92.17 ± 6.42^ab^	78.45 ± 9.49	76.90 ± 13.65		0.046
Aspartate		114.17 ± 11.18	94.76 ± 15.99	92.47 ± 22.28		0.093
Serine		86.20 ± 10.51^ab^	68.77 ± 13.04	60.46 ± 21.26		0.039
Glutamate		91.58 ± 5.94^b^	80.32 ± 8.38^b^	76.81 ± 12.17		0.039
Glycine		72.59 ± 21.57^b^	52.90 ± 19.37^b^	45.89 ± 19.78		0.038
Alanine		90.96 ± 8.36^ab^	75.96 ± 11.04	70.46 ± 17.46		0.043
Tyrosine		85.34 ± 12.77	68.20 ± 14.37	69.77 ± 18.14		0.147
Arginine		88.41 ± 7.95 ^ab^	74.53 ± 10.42	71.48 ± 14.21		0.047
Proline		87.21 ± 8.66 ^ab^	70.34 ± 13.21	64.14 ± 17.64		0.031
Cysteine		80.26 ± 12.11	60.03 ± 14.72	58.18 ± 22.03		0.079

* GM,phytase gene maize; PM, traditional parental maize of GM; ZD, commercialized maize “Zhengdan 958.”

**^#^**The digestibility of protein and amino acids was calculated by true digestibility and the digestibility of other nutrients was calculated by apparent digestibility.

a-significant vs PM group; b- significant vs ZD group.

### 3.4 Digestible Indispensable Amino Acid Score and Protein Digestibility Corrected Amino Acid Score

The DIAAS and PDCAAS of each amino acid are shown in Table 4. DIAAS is calculated by the digestibility of each essential amino acid, while PDCAAS is calculated by the true digestibility of protein. The final DIAAS and PDCAAS were the score of first limiting amino acids of each maize, which was used to evaluate and compare the protein quality of each maize strains. The final DIAAS of GM, PM, and ZD was 54.57, 31.75, and 33.91, respectively. The final DIAAS of GM was significantly higher than that of GM and PM. The final PDCAAS of GM, PM, and ZD were 58.94, 50.62, and 54.19, respectively. The final PDCAAS of GM was slightly higher than its parental maize, but no significant difference was found among groups. Both the result of DIAAS and PDCAAS showed that the limiting amino acids were lysine, ileucine, and methionine for all the experimental maize. The first limiting amino acid for GM, PM, and ZD was lysine.

**Table 4.** Comparison of DIAAS and PDCAAS of GM, PM and ZD*

Amino acids	Scoring model**	DIAAS(%)			PDCAAS(%)		
		GM	PM	ZD	GM	PM	ZD
Threonine	23	96.89 ± 23.15^b^	56.38 ± 25.95	51.89 ± 44.03	134.87 ± 16.22	103.53 ± 18.01	110.85 ± 28.90
Valine	39	88.65 ± 11.40	63.05 ± 13.45	71.05 ± 21.05	109.37 ± 13.15	82.30 ± 14.32	90.95 ± 23.71
Methionine	22	59.53 ± 7.82	45.85 ± 9.67	55.88 ± 15.40	70.50 ± 8.48	56.47 ± 9.83	65.50 ± 17.08
Isoleucine	30	59.02 ± 8.29^ab^	40.82 ± 10.49	40.70 ± 15.08	73.24 ± 8.81^ab^	55.22 ± 9.61	55.42 ± 14.45
Leucine	59	192.19 ± 14.32^ab^	159.13 ± 17.93	160.44 ± 29.91	219.08 ± 26.34	171.97 ± 29.92	174.72 ± 45.55
Phenylalanine	38	106.96 ± 9.66^ab^	83.00 ± 13.08	88.55 ± 17.79	125.85 ± 15.13	98.09 ± 17.07	99.18 ± 25.86
Lysine^#^	45	54.57 ± 7.55 ^ab^	31.75 ± 11.94	33.91 ± 16.79	68.94 ± 8.29	50.62 ± 8.81	54.19 ± 14.13
Tryptophan	6	105.23 ± 3.43	82.72 ± 5.93	103.00 ± 3.89	129.26 ± 15.54	86.28 ± 15.01	110.85 ± 28.90

*GM,phytase gene maize; PM, traditional parental maize of GM; ZD, commercialized maize “Zhengdan 958.”

** The scoring model for adult (aged above 18) revised by the FAO/WHO/UNU expert panel in 2007(WHO/FAO/UNU 2007)

^#^The first limiting amino acids

a-significant vs PM group; b- significant vs ZD group.

### 3.5 Blood Routines and Blood Chemistry

There was no significant difference in blood routines of BMPs before and after the digestibility experiment. Significant differences were found in ALT, ALP, TP and ALB. The ALT, TP and ALB were within the normal range of BMPs aged 10–11 months reported in literature.^40,41^ Although the ALB of BMPs was lower than reported in the literature, the level of ALT and AST were both in the normal range, which may not have special clinical significance (Table 5).

**Table 5.** Comparison of blood routine and biochemical results before and after the digestibility experiment

Specification	Before	After	p	Specification	Before	After	p
**Blood routines**							
WBC(10^9^/L)	15.98 ± 2.02	16.97 ± 3.91	0.596	PCT(%)	0.23 ± 0.06	0.24 ± 0.06	0.815
RBC(10^9^/L)	8.79 ± 0.28	8.44 ± 0.29	0.064	LYM(%)	62.07 ± 3.03	61.17 ± 1.58	0.534
HGB(g/L)	154.17 ± 6.40	148.83 ± 8.66	0.253	MON(%)	1.20 ± 0.09	1.08 ± 0.26	0.332
HCT(%)	0.46 ± 0.02	0.44 ± 0.02	0.080	NEU(%)	30.42 ± 2.46	32.68 ± 1.41	0.079
MCV(FL)	52.33 ± 1.86	51.83 ± 2.04	0.667	EOS(%)	4.55 ± 1.63	3.27 ± 0.41	0.091
MCH(pg)	17.55 ± 0.59	17.60 ± 0.88	0.910	BAS(%)	1.77 ± 0.41	1.80 ± 0.64	0.917
MCHC(g/L)	336.17 ± 2.64	339.00 ± 6.26	0.331	ALY(%)	3.68 ± 0.54	3.40 ± 0.91	0.528
RDW(%)	18.72 ± 1.25	17.58 ± 0.98	0.111	LIC(%)	0.28 ± 0.36	0.23 ± 0.15	0.757
PLT(10^9^/L)	279.67 ± 75.61	312.33 ± 87.11	0.504	IML(%)	0.15 ± 0.19	0.10 ± 0.06	0.549
MPV(FL)	8.27 ± 0.35	7.71 ± 0.62	0.088	IMG(%)	0.15 ± 0.18	0.13 ± 0.08	0.838
**Blood chemistry**							
ALT (U/L)	25.86 ± 5.29	34.65 ± 3.89	0.002	ALP(U/L)	43.03 ± 8.33	63.83 ± 8.59	0.000
AST (U/L)	40.47 ± 7.19	44.07 ± 1.95	0.128	BUN(mmol/L)	4.01 ± 0.87	3.62 ± 0.81	0.381
CRE(umol/L)	66.70 ± 3.94	67.88 ± 6.18	0.628	ALB(g/L)	27.57 ± 2.54	30.13 ± 1.97	0.047
TP(g/L)	60.08 ± 4.69	67.19 ± 3.85	0.006				

## 4. Discussion

Phytase is present in all types of organisms. Several phytase genes from plants, bacteria and fungi have been isolated and identified.^10^ Among them, the enzyme expressed by the phyA gene extracted from *Aspergillus Niger* can retain 25% of the activity in the animal digestive tract (average pH = 3.0). In 1998, Yao obtained the *phyA2* gene by modifying the *phyA* gene, and then expressed it in *Pichia pastoris* and used for large-scale fermentation to produce phytase.^42^ The activity of *phyA2* enzyme retained 40% in animal digestive tracts. After that, researchers have expressed the phytase genes from various *Aspergillus* species in different plants, such as wheat, rice, rape seeds etc.^10^ The phytase maize evaluated in this study was developed by the Chen Rumei team of the Chinese Academy of Agricultural Sciences from 2008. The exogenous gene *phyA2* was introduced in the traditional maize to reduce the combination of phytic acid and phosphorus to improve the utilization of phosphorus by increasing the phytase content in maize.

The expression product of exogenous *phyA2* gene is protein. Through components’ analysis, we found that there was no significant change in terms of protein and amino-acid contents. The content of protein and amino acids in GM was very close to its control materials (PM and ZD), and they were within the reference range of OECD and/or ILSI. The protein, fat, fiber, vitamins and minerals of GM, PM, and ZD are all within the normal range of OECD and/or ILSI. The carbohydrate content of GM was higher than that of ZD and PM, and it was within the reference value of ILSI and OECD. The minor differences may be due to natural variability, climatic conditions, soil conditions, and other factors. In general, we think the introduced *phyA2* does not affect the main nutritional composition of phytase maize comparing to its parental maize and the commercial available maize.

We adopted Bama miniature pig model to evaluate the digestibility of main nutrients in GM and the control materials, especially for protein. Bama miniature pig (BMP) is a high-quality breed and close group in China, which has the advantages of genetic stability, small size, low feeding requirements and strong disease resistance. The physiology, anatomy, drug metabolism, biochemical indexes, pathogenesis, etc., were very similar to that of human beings, and it has been used in cardiovascular, endocrine, and metabolism, digestion, stomatology, nervous system, and other medical fields. The structure of digestive system, the composition of intestinal flora and food digestion and absorption of BMPs are similar to that of human.^43^ Therefore, BMPs were ideal experimental animals to study nutrients’ digestibility. We selected ileal rather than fecal analysis method to determine nutrients’ digestibility in BMPs, because it reflects the actual digestion in the intestine, especially for protein/amino acids. The result of blood routines and chemistry showed that all BMPs were in normal physiological situation in the whole experimental period.

Phytate acts as an anti-nutrient in monogastric animals due to formation of stable complex with calcium, magnesium, copper, manganese, zinc and other metal ions and insoluble and unusable phytate protein, which would reduce the utilization of protein and minerals.^44,45^ As early as 1995, scientists had begun to add microbial phytase with different doses to pig’s feed to reduce the combination of phytic acid and protein, so as to promote the digestion and utilization of protein and amino acids.^46–48^ And they found the use of microbial phytase would increase the digestibility of amino acids in pigs. In our study, the phytatic acid phosphorus was decreased to 0.02 g/100 g in GM, comparing to 0.03 g/100 g in PM and 0.132 g/100 g in ZD. In addition, the proportion of phosphorus to total phosphorus in GM is as low as 6.92%, while that of parental maize was 10.38% and that of Zhengdan 958 was up to 52.73%. The phosphorus released by phytase in GM maize was slightly higher than that of PM and much higher than that of ZD In the digestibility experiment, although there is no significant difference in protein, fat and some amino acids, in general, the digestibility of protein, fat and amino acids of GM is still higher than ZD and PM, especially to ZD. The differences between individual BMP lead to a large standard deviation, which may be the main reason why the results are not statistically different. The ratio of phytate phosphorus to total phosphorus (10.38%) in PM was slightly higher than that of GM but much higher than that of ZD, however, the protein digestibility of PM did not improve significantly. According to the result of protein and amino acids composition analysis, the non-protein nitrogen compounds in PM was up to 15.30%, and that of ZD and GM was 11.74% and 8.20%, respectively. It might be one of the reasons for lower digestibility and DIAAS in PM. The digestibility was also influenced by pH and microbial proliferation at the site of the cannula reported by similar research with microbial phytase added.^46^ The contradiction between the content of phytate phosphorus and the protein/amino acids digestibility in PM might be a potential topic in future studies.

In order to obtain true ileal amino acid digestibility, we utilized 5% casein feed group to calculate the endogenous amino acid excretion rather than nitrogen-free feed. Zhang et al. found that most of the amino acid excretion in 5% casein feed group are higher than that of nitrogen-free feed group.^49^ Nitrogen-free feed used to be the most convenient method to determine the amount of endogenous amino-acid loss in pig ileum. However, the lack of protein in the daily feed will lead to negative nitrogen balance state and the decrease of intestinal digestive secretion, which would underestimate the intestinal endogenous amino acids loss. Therefore, many researchers think that 5% casein is the preferred method for determination of endogenous amino acid excretion, because it can stimulate on the digestive tract.^50,51^

The first limit amino acid of all the strains of maize is lysine, but the final DIAAS for PM and ZD are significantly lower than that of final PDCAAS (*P*< .05). PDCAAS used to be a typical method to analyze the protein digestibility since 1989. However, the PDCAAS does not adequately take into account the bioavailability of amino acids and it will overestimate protein quality of materials containing anti-nutritional factors.^37,52^ The results of our study also showed the defect of PDCAAS while evaluating the protein quality. In 2017, the FAO/WHO recommended a new and improved scoring system DIAAS to evaluate protein quality instead of PDCAAS.^37^ The calculation of DIAAS is based on the content of each amino acid in the test protein and the true ileal digestibility of each amino acid in the ileum. The DIAAS was recognized as the best method for diet protein quality assessment currently available in this field.^52^ However, the DIAAS is not widely reported to evaluate protein quality. In this study, we adopt the result of DIAAS to evaluate the protein quality in GM, PM, and ZD. The DIAAS of GM was significantly higher than that of PM and ZD by 71.8 and 60.9 percentage increase in lysine, respectively, which means the quality of protein in GM has been improved.

## 5. Conclusions

The present results suggest that the introduction of the *phyA2* gene in GM maize does not change the main nutrients nor the composition of amino acids, vitamins, and minerals. It does not interfere the digestion of protein/amino acid and has the ability to promote the digestion of amino acids by reducing the content of phytic acid. The protein quality of GM was significantly improved according to the result of DIAAS.

## Supplementary Material

Supplemental Material

## References

[1] Yang WZ, Pu LK, Zhang Q, Chen P, chen RM, Fan, YL . Stability of phytase in transgenic corn in simulated gastric/intestinal fluid. Chinese Journal of Agricultural Science and Technology. 2008;10(S1):86–89.

[2] Erpel F, Restovic F, Arce-Johnson P. Development of phytase expressing chlamydomonas reinhardtii for monogastric animal nutrition. BMC Biotechnol. 2016;16(1):1. doi:10.1186/s12896-016-0258-9. 26969115 PMC4788879

[3] Kumar V, Sinha AK, Makkar HPS, Becker K. Dietary roles of phytate and phytase in human nutrition: a review. Food Chem. 2010;120(4):945–59. doi:10.1016/j.foodchem.2009.11.052.

[4] Reddy CS, Vani K, Pandey S, Gupta G, Vijaylakshmi M, Reddy PCO, Kaul T . Manipulating microbial phytases for heterologous expression in crops for sustainable nutrition. Ann Plant Sci. 2013;2:436–54.

[5] Cowieson AJ, Ruckebusch JP, Sorbara JOB, Wilson JW, Guggenbuhl P, Tanadini L, Roos FF. A systematic view on the effect of microbial phytase on ileal amino acid digestibility in pigs. Anim Feed Sci Tech. 2018;231:138–49. doi:10.1016/j.anifeedsci.2017.07.007.

[6] Yu CH, Tian ST, Lu XB, Li F, Yang SK, Sun HW . Qualitative and quantitative PCR detection of phytase gene (phya2) in Maize. Chinese Journal of Agricultural Biotechnology. 2012;4:356–61.

[7] Chen L, Wang T. Effect of Phytase on energy, protein and amino acid utilization and digestive enzyme activity of Cherry Valley Duck. Acta Zoonutrimenta Sinica. 2009;6:146–52.

[8] Richardson AE, Hadobas PA, Hayes JE. Extracellular secretion of Aspergillus phytase from Arabidopsis roots enables plants to obtain phosphorus from phytate. The Plant Journal. 2001;25(6):641~649. doi:10.1046/j.1365-313x.2001.00998.x. 11319031

[9] Zhang Q, Chen RM, Yang WZ, Chen P, Xue GX, Fan YL . The obtaining of transgenic maize plants with PhyA2 gene constitutive express phytase. Journal of Agricultural Biotechnology. 2010;18(4):623~629.

[10] Chen RM, Xue GX, Chen P, Yao B, Yang W, Ma Q, Fan Y, Zhao Z, Tarczynski MC, Shi J, et al. Transgenic maize plants expressing a fungal phytase gene. Transgenic Res. 2008;17(4):633–43. doi:10.1007/s11248-007-9138-3. 17932782

[11] Chinese ministry of agriculture. Approval list of agricultural genetically modified organisms safety certificate in 2014. Retrived from http://www.moa.gov.cn/ztzl/zjyqwgz/spxx/201506/P020160524354739618071.pdf (accessed on November 28, 2020).

[12] Chinese ministry of agriculture. Approval list of agricultural genetically modified organisms safety certificate in 2009. http://www.moa.gov.cn/ztzl/zjyqwgz/spxx/201202/P020120203355472286958.pdf (accessed on November 28, 2020).

[13] Zhang Y, LingHu LQ, Hu YC, Yang XG, Piao JH, Zhang YP, Yang LC . Nutritional evaluation and analysis of AO transgenic high phytase maize. China Food and Nutrition. 2017;23(5):50–54.

[14] Xiong JW, Peng D, Tan XJ, Wang JR . Research and safety evaluation of phytase transgenic maize. Genomics and Applied Biology. 2011;30(2):251-256.

[15] Li XH. Overcome technical difficulties and resolutely win the turnaround in the corn seed industry. China Agricultural Information Network, Information Center of the Ministry of Agriculture and Rural Affairs. Accessed on July 6, 2021.

[16] Chinese National Bureau of Statistics. National data-food production 2020, Accessed on Nov 1, 2021.

[17] Chinese national standard GB/T 6432-2018. Determination of crude protein in feed, Standards Press of China, Beijing (China).

[18] Chinese national standard GB/T 6433-2006 2006. Determination of fat in feed, Standards Press of China, Beijing (China).

[19] Chinese national standard GB/Z 21922-2008, 2008. Basic terms of food nutrients. Standards Press of China, Beijing (China).

[20] Chinese national standard GB/T 6434-2006. Determination of crude fiber content in feed- filtration method, Standards Press of China, Beijing (China).

[21] Chinese national standard GB/T 6435-2014. Determination of water in feed, Standards Press of China, Beijing (China).

[22] Chinese national standard GB/T 6438-2007. Determination of crude ash in feed, Standards Press of China, Beijing (China).

[23] Chinese national standard GB 5009.124-2016. Determination of amino acids in foods, Standards Press of China, Beijing (China).

[24] Chinese national standard GB 5009.82-2016. Determination of vitamin A, vitamin D and vitamin E in foods, Standards Press of China, Beijing (China).

[25] Chinese national standard GB 5009.84-2016. Determination of vitamin B1 in foods, Standards Press of China, Beijing (China).

[26] Chinese national standard GB 5009.85-2016. Determination of vitamin B2 in foods, Standards Press of China, Beijing (China).

[27] Chinese national standard GB 5009.154-2016. Determination of vitamin B6 in foods, Standards Press of China, Beijing (China).

[28] Chinese national standard GB 5413.14-2010. Determination of vitamin B12 in infant food and dairy products, Standards Press of China, Beijing (China).

[29] Chinese national standard GB 5009.89-2016. Determination of niacin and nicotinamide in food, Standards Press of China, Beijing (China).

[30] Chinese national standard GB/T 13088-2006. Determination of chromium in feed, Standards Press of China, Beijing (China).

[31] Chinese national standard GB 5009.268-2016. Determination of multi elements in food, Standards Press of China, Beijing (China).

[32] Chinese national standard GB 5009.87-2016. Determination of phosphorus in food, Standards Press of China, Beijing (China).

[33] Hu YC, Li M, Piao JH, Yang X. Nutritional evaluation of genetically modified rice expressing human lactoferrin gene. J Cereal Sci. 2010;52(3):350–55. doi:10.1016/j.jcs.2010.05.008.

[34] Yang YX, Ge KY. Encyclopedia of nutrition science. Beijing (China): People’s Health Press; 2019.

[35] Leser S. The 2013 FAO report on dietary protein quality evaluation in human nutrition: recommendations and implications. Nutr Bull. 2013;38(4):421–28. doi:10.1111/nbu.12063.

[36] WHO/FAO. Report of a Joint FAO/WHO expert consultation: protein quality evaluation. FAO Food & Nutrition Paper, 1991; 51:1. 1817076

[37] WHO/FAO/UNU. Protein and amino acid requirements in human nutrition. Report of a Joint WHO/FAO/UNU Expert Consultation, Geneva, WHO Technical Report Series, 2017; 935.

[38] OECD. Consensus document on compositional considerations for new varieties of maize (Zea mays): key food and feed nutrients, anti-nutrients and secondary plant metabolites. Organization for Economic Cooperation and Development, Paris (France), 2002.

[39] ILSI . International life sciences institute crop composition database, Version 7.0, www.cropcomposition.org (accessed in Feb28,2020).

[40] Yang LG, Ding J, Zhou CX, Ren BM, Xie D, Yang WM . Comparison of blood physiological and biochemical indexes between two groups of Bama minipigs. Laboratory Animal and Coparative Medicine. 2015;35(3):230–33.

[41] Yang SL, Ren HY, Wang H, Feng ST, Wang AD, Gan SX, Li K . Analysis of blood biochemical indexes of three experimental miniature pig breeds in China. China Animal Husbandry & Veterinary Medicine. 2007;34(4):75–78.

[42] Yao B, Zhang CY, Wang JH, Fan YL. Recombinant Pichia pastoris overexpressing bioactive phytase. Sci China C. 1998;41(3):330–36. doi:10.1007/BF02895110. 18425641

[43] Zou DS, Yu J. Research progress on animal models of Bama miniature pigs in the field of medcine. China Animal Husbandry & Veterinary Medicin. 2017;44:1128–34.

[44] Guggenbuhl P, Nunes S. Effects of two phytases on the ileal apparent digestibility of minerals and amino acids in ileo-rectal anastomosed pigs fed on a maize-rapeseed meal diet. Livest Sci. 2007;109(1–3):0–263. doi:10.1016/j.livsci.2007.01.110.

[45] Reddy CS, Kim SC, Kaul T. Genetically modified phytase crops role in sustainable plant and animal nutrition and ecological development: a review. Biotech. 2017;7(3):195. doi:10.1007/s13205-017-0797-3. PMC5493567 28667635

[46] Cowieson AJ, Ruckebusch JP, Sorbara J, Wilson JW, Guggenbuhl P, Roos FF. A systematic view on the effect of phytase on ileal amino acid digestibility in broilers. Anim Feed Sci Tech. 2017;225:182–94. doi:10.1016/j.anifeedsci.2017.01.008.

[47] Zouaoui M, Létourneau-Montminy MP, Guay F. Effect of phytase on amino acid digestibility in pig: a meta-analysis. Animal Feed Science and Technology. 2018;238:18–28. doi:10.1016/j.anifeedsci.2018.01.019.

[48] Hong B, Kim BG. Supplemental phytase increases phosphorus digestibility in pigs regardless of phytase source or feed pelleting. Animal Feed Science and Technology. 2021;276:114901. doi:10.1016/j.anifeedsci.2021.114901.

[49] Zhang YC, Li DF, Fan SJ, Piao X, Wang J, Han IK. Effects of casein and protein-free diets on endogenous amino acid losses in pigs. Asian Austral J Anim. 2002;15(11):1634–38. doi:10.5713/ajas.2002.1634.

[50] Cervantes-Pahm SK, Stein HH. Ileal digestibility of amino acids in conventional, fermented, and enzyme-treated soybean meal and in soy protein isolate, fish meal, and casein fed to weanling pigs. J Anim Sci. 2010;88(8):2674–83. doi:10.2527/jas.2009-2677. 20407072

[51] van Kempen TA, Kim IB, Jansman AJ, Verstegen MW, Hancock JD, Lee DJ, Gabert VW, Albin DM, Fahey GC Jr, Grieshop CM *et al* . Regional and processor variation in the ileal digestible amino acid content of soybean meals measured in growing swine. J Anim Sci. 2002;80(2):429–39. doi:10.2527/2002.802429x. 11883431

[52] Wolfe RR, Rutherfurd SM, Kim IY, Moughan PJ. Protein quality as determined by the digestible indispensable amino acid score (DIAAS): evaluation of factors underlying the calculation. Nutr Rev. 2016;74(9):584. doi:10.1093/nutrit/nuw022. 27452871 PMC6322793

