# Supplementary material for: Nutritional components and protein quality analysis of genetically modified phytase maize
Source: GM Crops Food. 2022 Feb 1;13(1):15–25. doi: 10.1080/21645698.2021.2009418 (PMC8890400; doi:10.1080/21645698.2021.2009418)
Supplement: Supplemental Material [file KGMC_A_2009418_SM1828.zip › Appendix 1.docx]

**Appendix 1**

**Table S1** The diet formula of digestibility experiment^*^

| Components (g) | GM | PM | ZD | 5% casein |
| --- | --- | --- | --- | --- |
| Experimental corn | 938.4 | 938.1 | 937.3 | 0 |
| casein | 0 | 0 | 0 | 50 |
| sucrose | 0 | 0 | 0 | 200 |
| corn starch | 0 | 0 | 0 | 50 |
| fiber | 0 | 0 | 0 | 632.9 |
| vegetable oil | 10 | 10 | 10 | 10 |
| salt | 5 | 5 | 5 | 5 |
| chromium trioxide | 5 | 5 | 5 | 5 |
| 1% premix | 10 | 10 | 10 | 10 |
| calcium monophosphate^1^ | 21.3 | 21.9 | 23.9 | 34 |
| stone powder^2^ | 10.3 | 10 | 8.8 | 3.1 |
| total | 1000 | 1000 | 1000 | 1000 |
| Nutrient levels |  |  |  |  |
| protein（%） | 7.93% | 8.01% | 7.63% | 4.58% |
| Total phosphorus（%） | 0.62 | 0.63 | 0.67 | - |

^*^GM, genetically modified maize with phytase gene; PM, parental maize of GM; ZD, zhengdan 958, the most popular and commercialized maize strain in China;

^1^ calcium monophosphate: phosphorus 17.62%, calcium 20.35%;

^2^ stone powder: calcium 35%

**Table S2** Formula of 1% premix

| Premix | component(g) |
| --- | --- |
| Vitamin complex* | 4 |
| Mineral complex# | 20 |
| choline | 16 |
| middling | 20 |
| non transgenic vegetable oil | 1 |
| corresponding maize | 39 |
| Total | 100 |

*The formula was showed in table 3; # The formula was showed in table 4

**Table S3** Formula of vitamin complex

| Vitamin | Specifications | component(g) |
| --- | --- | --- |
| vitamin A | 5,000,000 IU/g | 7.5 |
| vitamin D3 | 5,000,000 IU/g | 0.50 |
| vitamin E | 50% | 25.00 |
| vitamin B1 | 96% | 2.60 |
| vitamin B2 | 80% | 6.25 |
| vitamin B6 | 98% | 3.06 |
| vitamin B12 | 1% | 2.00 |
| biotin | 2% | 5.00 |
| Pantothenic acid | 98% | 7.65 |
| folic acid | 98% | 1.02 |
| nicotinic acid | 99% | 15.15 |
| vitamin k3 | 94% | 1.33 |
| corresponding maize |  | 22.94 |
| Total |  | 100 |

**Table S4** Formula of mineral complex

| Minerals | Specifications(%) | component(g) |
| --- | --- | --- |
| Ferrous sulfate monohydrate | 92 | 39.85 |
| Cupric sulfate monohydrate | 94 | 8.94 |
| Manganese sulfate monohydrat | 98 | 14.35 |
| Zinc Sulphate Monohydrate | 95 | 11.28 |
| potassium iodide | 1 | 13.09 |
| Sodium selenite | 1 | 3.29 |
| corresponding maize |  | 9.20 |
| Total |  | 100 |
