# Supplementary material for: Nutritional components and protein quality analysis of genetically modified phytase maize
Source: GM Crops Food. 2022 Feb 1;13(1):15–25. doi: 10.1080/21645698.2021.2009418 (PMC8890400; doi:10.1080/21645698.2021.2009418)
Supplement: Supplemental Material [file KGMC_A_2009418_SM1828.zip › Appendix 2.docx]

**Appendix 2**

Table S5 The Experimental arrangement

| Experiment days | Animal number and arrangement | | | | |
| --- | --- | --- | --- | --- | --- |
|  | 1-2 | 3-4 | | 5-6 | 7-8 |
| 1-6 | GM adaptation | ZD adaptation | | PMadaptation | Casein adaptation |
| 7-9 | GMchyme collection | ZD chyme collection | | PMchyme collection | Casein chyme collection |
| 10-15 | Normal commercial diet | | | | |
| 16-21 | Casein adaptation | GM adaptation | | ZD adaptation | PMadaptation |
| 22-24 | Casein chyme collection | GMchyme collection | | ZD chyme collection | PMchyme collection |
| 25-30 | Normal commercial diet | | | | |
| 31-36 | PM adaptation | Casein adaptation | GM adaptation | | ZD adaptation |
| 37-39 | PMchyme collection | Casein chyme collection | GM chyme collection | | ZD chyme collection |
| 40-45 | Normal commercial diet | | | | |
| 46-51 | ZD adaptation | PM adaptation | Casein adaptation | | GM adaptation |
| 52-54 | ZD chyme collection | PMchyme collection | Casein chyme collection | | GM chyme collection |
| 55-60 | Normal commercial diet | | | | |

GM, genetically modified maize with phytase gene; PM, parental maize of GM; ZD, zhengdan 958, the most popular and commercialized maize strain in China;
